# Characterizing Depression Issues on Sina Weibo

**DOI:** 10.3390/ijerph15040764

**Published:** 2018-04-16

**Authors:** Xianyun Tian, Philip Batterham, Shuang Song, Xiaoxu Yao, Guang Yu

**Affiliations:** 1 School of Management, Harbin Institute of Technology, Harbin 150001, China; uncertainorcertain@gmail.com (X.T.); songshuangfighting@gmail.com (S.S.); xxyao06@gmail.com (X.Y.); 2 Centre for Mental Health Research, The Australian National University, Canberra ACT 2601, Australia; philip.batterham@anu.edu.au

**Keywords:** social media, mental health, public health, depression, Sina Weibo

## Abstract

The prevalence of depression has increased significantly over the past few years both in developed and developing countries. However, many people with symptoms of depression still remain untreated or undiagnosed. Social media may be a tool to help researchers and clinicians to identify and support individuals who experience depression. More than 394,000,000 postings were collected from China’s most popular social media website, Sina Weibo. 1000 randomly selected depression-related postings was coded and analyzed to learn the themes of these postings, and a text classifier was built to identify the postings indicating depression. The identified depressed users were compared with the general population on demographic characteristics, diurnal patterns, and patterns of emoticon usage. We found that disclosure of depression was the most popular theme; depression displayers were more engaged with social media compared to non-depression displayers, the depression postings showed geographical variations, depression displayers tended to be active during periods of leisure and sleep, and depression displayers used negative emoticons more frequently than non-depression displayers. This study offers a broad picture of depression references on China’s social media, which may be cost effectively developed to detect and help individuals who may suffer from depression disorders.

## 1. Introduction

With 503 million users, Sina Weibo is the most popular social media website in China [1]. It can be considered as the Chinese equivalent of Twitter. Like Twitter, Weibo allows its users to post updates and these postings can be seen by users who follow them. About 100 million messages are posted on it every day. These postings cover many different themes, including news, product promotions, shared documents, and users’ opinions, so users can acquire different kinds of information from Sina Weibo [2]. For researchers, these data offer an opportunity to better understand the community and society. For example, some researchers have explored the potential of social media to provide real-time physical activity surveillance [3]. Other researchers have attempted to examine Twitter’s predictive potential of consumer purchasing by observing the relationship between Twitter posts and stock market activity [4]. Due to the anonymity of social media, people often discuss private issues on it, including physical illness and mental disorders, which may be difficult to discuss face-to-face because of social or cultural factors [5,6,7,8,9,10]. 

Depression is one of the most burdensome diseases in the world [11]. It is projected to be the leading cause of disability over the next 20 years [11]. Depression prevalence has increased significantly over the past years both in developed and developing countries [12]. However, even in the United States where many primary care programs have been devised to manage depression in the community, many people who meet depression criteria still remain untreated or undiagnosed [13]. Fortunately, social media may be a tool to help researchers and clinicians to identify and support individuals who experience depression, as people with mental disorders may use social media to communicate with others to acquire health-related information or support or disclose their experiences [2,5,14,15].

Existing research has explored the feasibility of using social media to identify and understand the individuals who report physical illness or mental disorders. For example, researchers have investigated sleep complaints on social media to identify the individuals who may suffer from insomnia [5,16,17]. People living with HIV (Human Immunodeficiency Virus) have also been studied using social media to learn their emotional state and needs [6,18]. Previous research has also investigated depression symptoms and suicide risk using social media [19,20,21,22,23]. For example, associations between references to depression symptoms on Facebook and self-reported depression symptoms using a clinical screen have been established [19]. Preferred approaches for supporting young people who disclose depression on Facebook have also been examined [20]. In addition, a content analysis was conducted to assess the attitudes of mass media organizations and public opinion leaders toward depression [24]. 

Previous studies have shown that the correlation between symptoms of addictive technology use and mental disorder symptoms was positive and significant [25,26]. This, from another perspective, suggests that it may be a good way to identify individuals who may suffer from mental disorders based on social media use.

Depression is a very costly mental disorder in China. It is reported that the total estimated cost of depression in China was more than US$ 6 billion in 2002, which represents an enormous economic burden [27]. However, research focusing on using social media to investigate China’s depression issues is still in its infancy. More research is needed to help health professionals, governments, and researchers to better understand the characteristics of people who disclose depression their on Sina Weibo. Existing research mainly focuses on studying depression using Twitter or Facebook [10,15]. However, these two sites cannot be used to study China’s depression issues, because they are not accessible in Mainland China. Although expressions of depression on Sina Weibo have been investigated [24], the feelings, experiences, and emotions of the individuals who report depression have not been identified. Additionally, although the feasibility of using social media to predict depression has been validated [28], the demographic characteristics of the individuals who display references to depression have not been adequately studied. Besides, their model only identified active users and users who tended to fill the Center for Epidemiologic Studies Depression Scale (CES-D) [28].

To fill the aforementioned gaps in the research, this study was undertaken with the aim of identifying and describing the people who may experience depression using social media, so that better detection and intervention measures can be adopted to assist them. Specifically, the intention was to determine the themes of depression-related postings; whether it is feasible to extract the postings from whence depression is reported; and the demographic characteristics, diurnal activity patterns, and geographic distribution of the individuals who report depression.

## 2. Methods

### 2.1. Data Collection

More than 394,000,000 postings were collected from Sina Weibo through its API (Application Programming Interface) from 17 May to 21 August 2015. These postings belonged to one million users who were randomly sampled from Sina Weibo. For every user in this sample, his/her latest 1000 postings were collected, and if the total number of his/her postings was fewer than 1000, then all of his/her postings were sampled. The reason for obtaining 1000 postings for each user was that the number of most users’ postings was less than 1000. In addition to the postings, other variables, such as demographic information, the number of followers, postings, and following were also obtained from Sina Weibo.

**Figure 1:**
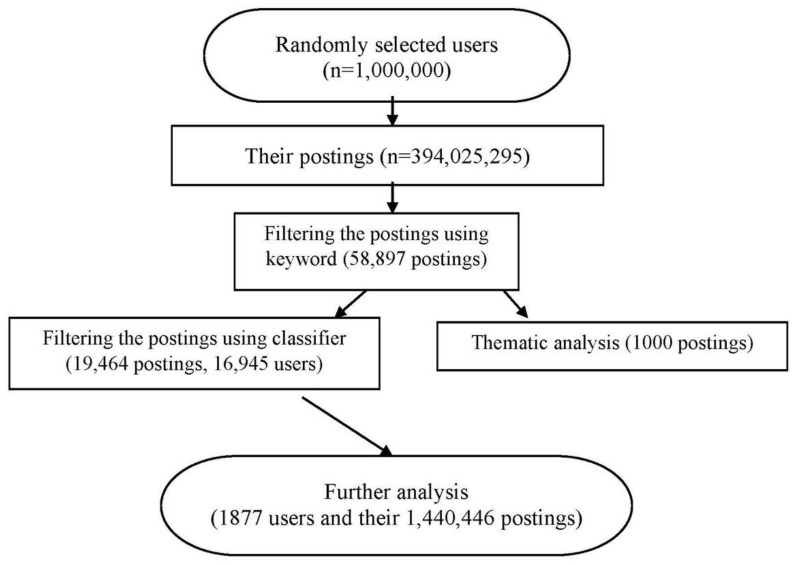
Flowchart of procedure for selecting postings related to depression.

All the raw data used in this study were publicly available. However, to protect users’ privacy, only aggregate information is reported. The flow chart of the data processing is presented in Figure 1.

### 2.2. Thematic Analysis

To identify the themes of the postings in which people mentioned depression, all the postings that contained the keyword “depression” (“抑郁”in Chinese) were retrieved. The reason for not including other keywords, such as “feeling down” (“心情低落” in Chinese) or “unhappy” (“不开心” in Chinese), was because these keywords were not depression-specific enough, and including them would introduce noisy information. Eventually, 58,897 postings were found.

From the 58,897 postings, 1000 were randomly selected and the themes within them identified. Two research team members were trained to independently read the 1000 postings to determine the most popular themes and create a codebook. Themes were defined as topics that occur or reoccur [29]. Identified themes of the postings were as follows: (1) disclosure of depression; (2) philosophical thoughts on life; (3) shared medical information; (4) news; (5) advertisement; (6) help seeking; and (7) ambiguous postings.

After coding, each of the 1000 postings was discussed by the two research members in order to reach an agreement on the final assigned code. A third member of the research team was trained to code 100 postings randomly selected from the 1000 postings to check the inter-coder reliability. Inter-coder reliability for each theme was as follows: (1) disclosure of depression—agreement 87%, kappa 0.74; (2) philosophical thoughts on life—agreement 91%, kappa 0.68; (3) shared medical information—agreement 95%, kappa 0.84; (4) news—agreement 100%, kappa 1.00; (5) advertisement—agreement 94%, kappa 0.23; (6) help-seeking—agreement 91%, kappa 0.48; (7) ambiguous posting—agreement 96%, kappa 0.70.

### 2.3. Building a Classifier

The thematic analysis suggests that not all postings containing the keyword depression can be utilized to identify individuals who may be experiencing depression. Hence, it was necessary to build a text classifier through which postings indicating depression could be found. A text classifier is a model that can distinguish one kind of text from others. In this study, a text classifier was built to identify the disclosure of depression from other kinds of postings.

A labeled dataset is required to train a text classifier [30]. However, to the researchers’ knowledge, a dataset meeting this requirement is not publicly available. Consequently, three research team members independently labeled 5000 postings that were randomly selected from the 58,897 postings. The Majority-Rule was adopted to determine the assigned label when there was disagreement. Three kinds of machine learning algorithms, including Logistic Regression [31], Random Forest [32], and Support Vector Machine [33], were selected to train the classifier based on our labeled dataset. Ten-folded cross validation was adopted to compute the accuracy of every algorithm.

The experimental results suggest that SVM (Support Vector Machine) performed best in this task and obtained an accuracy of 86%. Thus, the SVM classifier was applied to identify target postings. Eventually, 19,464 postings belonging to 16,945 users were classified as target postings. To improve the likelihood that users were indeed discussing depression rather than chance comments unrelated to the user’s experience, we retained only the users who posted more than one depression posting. After selecting users with multiple postings, 1877 users were identified for further analysis.

### 2.4. Demographic Characteristics

The 1877 identified users’ demographic information was retrieved from our database. For comparison, the demographic information of a randomly selected comparsion group was also obtained. The size of the comparsion group was 910,000. The demographic information included gender, the location of users, the number of followers, the number of following, the number of postings, and so on.

Pearson’s chi-square test with Yates’ continuity correction was applied to determine whether there was a statistically significant difference in gender distribution between users with depression references and the general population. The Wilcoxon Rank Sum and Signed Rank Test [34] was adopted to determine whether there was a statistically significant difference in the distributions of followers, following, and postings between users with and without depression postings. Cohen’s d was employed to calculate the effect sizes between depression displayers and the general population on the number of following, postings, and followers. *p* < 0.001 was considered statistically significant. 

To estimate national and regional prevalence of users with depression references in China, the number of users with depression postings was divided by the population in each area.

### 2.5. Diurnal Patterns

The messages posted by depression displayers were retrieved from our database to understand the diurnal pattern of these users’ online behavior. The total number of their postings was 1,440,466. For comparison purposes, 1,000,000 postings posted by the comparison group (also called general population) were randomly selected from our database. The percentage of messages posted per hour was calculated as the metric to measure diurnal patterns.

To understand the difference in the distribution of postings between the two groups, each day was divided into three periods: sleep hours, work hours, and leisure hours. The number of postings in each period posted by the two groups was calculated, and Pearson Chi-square was employed to learn whether the difference between the two groups was statistically significant based on *p* < 0.001.

### 2.6. Patterns of Emoticon Usage

Since emoticons are widely used to express individuals’ feelings, it is reasonable to expect that the usage of emoticons between depression displayers and the general population may be different. We examined the most frequently used ten emoticons within the two groups. The percentage of negative emoticons (based on the ten most popular) was calculated. A Pearson’s Chi-squared test with Yate’s continuity correction [35] was applied to determine whether the difference between the two groups was statistically different.

## 3. Results

### 3.1. Thematic Analysis

From the 1000 selected postings, themes were extracted to learn what themes were discussed when depression was mentioned on social media. The themes of the selected postings are shown in Table 1.

Disclosure of depression was the most common theme. This suggests that it may be feasible to identify the individuals who may suffer from depression using a keyword-based method. About 20% of the postings were shared medical information about prevention, diagnosis, and treatment of depression. However, the majority of these postings were posted by ordinary users, not professionals, celebrities, or organizations. Philosophical thoughts on life that were related to depression (15.8%) were also discussed. Only 10.1% of the postings mentioned help-seeking on social media. The rest of the postings were news (2.4%), and advertisements or ambiguous postings that could not be classified (12.4%). The latter two are not included in the table, since they did not provide useful information about depression.

### 3.2. Demographic Characteristics

To understand the pattern of demographic characteristics of the individuals who disclosed depression on Sina Weibo, we examined their online characteristics, including gender, followers, following, and messages. The results are shown in Table 2. The results suggest that females (79.9%) tended to report depression on social media more commonly than males (20.1%). This result is consistent with previous research indicating that the prevalence of clinical depression is higher in females than males [36,37,38].

Compared to typical Sina Weibo users, the depression displayers had more postings within the study period. However, there was no statistically significant difference between the two groups on the number of following and followers.

**Figure 2:**
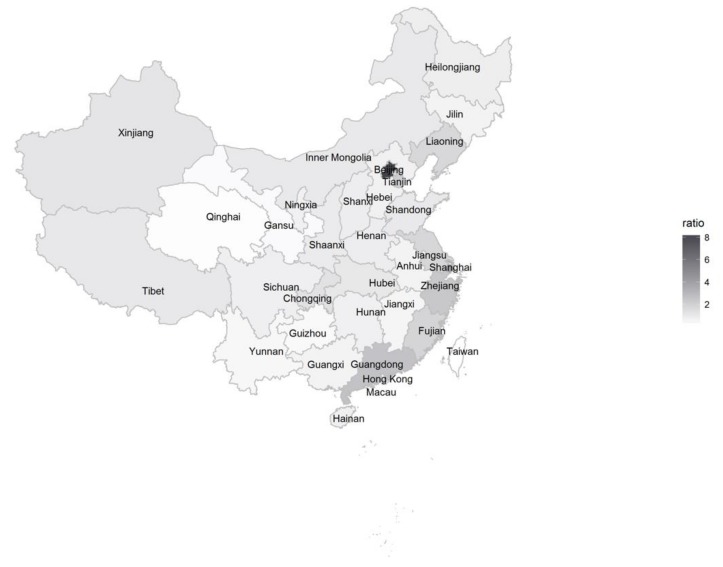
Geographic distribution of users with depression.

Figure 2 suggests that more individuals mentioned depression in the southeast regions of China. These regions including Zhejiang, Guangdong, Fujian, and Jiangsu, are the most developed economic areas in China. The two largest cities, Beijing and Shanghai, also had a high prevalence of depression posts.

### 3.3. Diurnal Patterns

**Figure 3:**
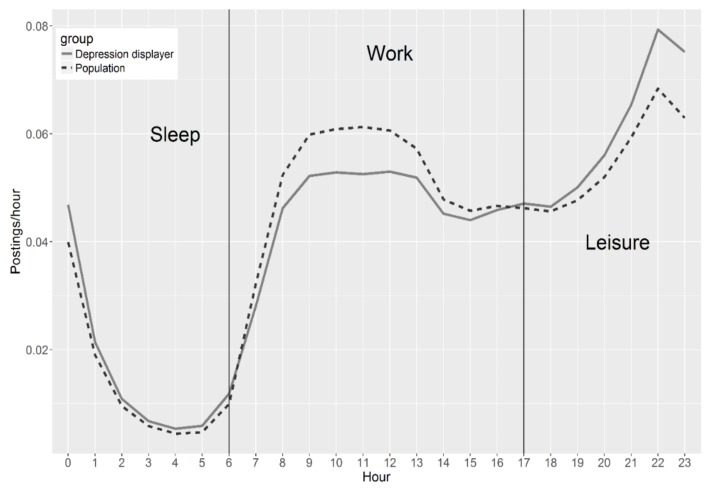
Diurnal trends (average number of postings posted hourly throughout a day).

The result of Pearson Chi-square suggested that the difference between two groups on distribution of postings was statistically significant. Figure 3 depicts the diurnal patterns of the postings of the two groups. Figure 3 shows that the depression displayers tended to be more active during leisure and sleep period compared to typical Sina Weibo users.

### 3.4. Pattern of Emoticon Usage

Emoticons are commonly on Sina Weibo to express feelings, positive or negative. To examine whether there were differences of emoticon usage between the two groups, we examined the ten most frequently used emoticons. The results are shown in Table 3.

The result indicates that eight of the ten emoticons were shared by the two groups. Surprisingly, the most frequently used emoticon by the non-depressive users was also a negative one. Five different negative emoticons were used among the top ten by the depression group, compared to the three among non-depressive users. The percentage of total negative emoticons used by the depression group was 57.2%, while among the non-depressive users 39.3% were negative. There was a statistically significant difference (χ^2^ = 62.7, *p* < 0.001) between the two groups.

## 4. Discussion

This study investigated users who reported depression on Sina Weibo to identify the themes of depression-related postings and the characteristics of these individuals. Through content analysis, it was found that the most common theme was the disclosure of depression, which supports the feasibility of using social media to identify and support individuals experiencing depression. Shared medical information was the second most popular theme of the depression-related postings. This sharing of information may be positive, reflecting that people are paying more attention to depression in China. However, some sources of the medical information may not be evidence-based, resulting in potential risk to users on social media; further investigation of the veracity of medical information is therefore warranted. In addition, 10% of the postings were about help-seeking, suggesting that social media has been used as a tool by people experiencing depression symptoms to seek help from others. 

This study also characterized individuals who displayed references to depression on Sina Weibo. Previous research on Sina Weibo has mainly concentrated on building a model to predict individuals who may be experiencing depression [28]. However, our study provides a snapshot of the demographic characteristics of these people. The results suggest that females tend to report depression on social media more than males. It is true that this result is consistent with previous findings. However, this larger gender difference in depression postings is not readily explainable by disparities in diagnosis of depression. One possible explanation is that females are more likely to express their feeling than males. The results also suggest that depression displayers tend to be more active on Sina Weibo than the general population. This pattern suggests that depression displayers may be more dependent on social media than the general population. However, this pattern is inconsistent with findings from Twitter [39], suggesting that people with depression have lower numbers of followers, following, and decreased desire for social interaction. The platform or culture may cause this difference. Additional research is required to provide a better understanding of this finding.

The diurnal activity analysis suggests that depression displayers tend to be more active during leisure and sleep time. It is not surprising that depression displayers tend to be more active during sleep time, because the individuals who suffer from depression are often bothered by insomnia. Previous research has found that people who suffer from insomnia tend to be more active than non-depressive users during sleep periods [5]. However, more research is required to determine why they are also more active during leisure time and less active in work hours. One possible explanation may be that depression displayers were younger on average and tended to more active during sleep and leisure period. Further research is needed to determine whether this is true, because most users’ age information was not accessible on social media. It may be most effective to deliver tailored information to these individuals when they are most active. Further research is required to explore the time of day when information or support would be most helpful.

Geographical variations in depression posts were observed. A possible reason that the individuals who lived in rich areas, including big cities, tended to express depression may be that the people living in these areas experience fiercer competition and greater social fragmentation. Another reason may be that people living in these areas are knowledgeable about depression, which resulted in disclosure of depression. More research is required to investigate the reason behind this finding. Our discovery on the geographic distribution of depressed users may be helpful in assisting the Chinese government to distribute health resources efficiently, given its large population and limited resources.

It is worth noting that identified users in this study were not clinically diagnosed. Some of them might also be high on neuroticism just like the users identified in an anxiety-related study [40]. People who score high tend to express more feelings of anxiety or depression than average [41,42].

There were some limitations of our study. The depression-related postings were collected using a keyword-based method, which is efficient but not highly accurate. Postings that may have also been related to depression but did not contain the particular keyword were missed. This omission may have resulted in a sampling bias, which means that the individuals identified were only the people who explicitly mentioned depression in their postings. Consequently, regional variations may reflect knowledge of depression.

It should be noted that the method used in this study is not a substitute for traditional clinical diagnosis. It is, however, an effective complement to the traditional methods because of its low cost in identifying and assisting potential depression sufferers. Intervention programs could be developed and delivered based on our findings. For example, some tailored programs could be developed to encourage the people who disclosed depression on social media to seek help from professional facilities.

## 5. Conclusions

Despite its limitations, our study offers a broad picture of depression references on China’s social media. Many people openly posted about depression on Sina Weibo. Disclosure of depression was the most prevalent theme among the collected postings. Medical information about depression was frequently shared. Additionally, we found that the depression displayers tended to live in the affluent areas of China and display particular demographic characteristics and diurnal patterns that tended to be consistent with existing epidemiological and clinical data on depression. Our findings may be used to help mental health services and the Chinese government to better understand the prevalence and correlates of depression in China. The findings may also be used to develop more efficient measures to support people experiencing depression.

## Figures and Tables

**Table 1:** Themes of depression-related postings.

Themes	*N* (%)	Sample Postings
Disclosure of depression	388 (38.8%)	Nothing I feel depressed.
Philosophical thoughts on life	158 (15.8%)	Two tragedies of life: having too high or too low expectations. People living in this modern world are too vulnerable. It has been reported that many celebrities have experienced depression. These people must have undergone tremendous changes in self-awareness. They used to believe they were omnipotent. However, setbacks make them feel inferior when they occur.
Shared medical information	205 (20.5%)	A research team from the University of Pittsburgh demonstrated that taking more fatty acid that is good for your body can reduce the risk of depression. Meat always makes you feel bad. However, fish can keep bad moods away.
Help seeking	101 (10.1%)	I feel sorrowful, is this a sign of depression?
News	24 (2.4%)	Fewer tourists visit Australia because of the hurricane. A wombat named Tonka is suffering from depression from getting no cuddles. He/she has lost 20% of his/her weight. How pathetic he/she is!

**Table 2:** Demographic characteristics of Sina Weibo users.

Demographic Variables	Depression	Population	Effect Size	*p*
Gender				<0.001
Male	20.1%	43.8%		
Female	79.9%	56.2%		
Following			0.016	<0.001
25%	91	75		
Median	169	147		
75%	282	260		
Followers			0.005	<0.001
25%	111	62		
Median	198	133		
75%	427.9	270		
Postings			0.201	<0.001
25%	477	94		
Median	1126	296		
75%	1258	751		

**Table 3:** Emoticons used by the two groups.

Depression Displayers			Non-Depressive Users		
Emoticon	Percent	Label	Emoticon	Percent	Label
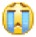	29.05%	Negative	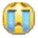	23.19%	Negative
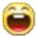	12.95%	Positive	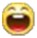	15.54%	Positive
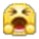	12.76%	Negative	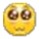	11.06%	Positive
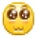	10.23%	Positive	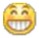	10.69%	Positive
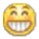	8.56%	Positive	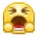	10.05%	Negative
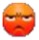	6.86%	Negative	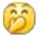	7.31%	Positive
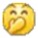	6.17%	Positive	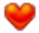	6.79%	Positive
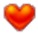	4.89%	Positive	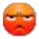	6.09%	Negative
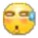	4.76%	Negative	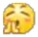	5.40%	Positive
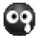	3.79%	Negative	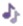	3.87%	Positive
